# Risk factors of falls among elderly living in Urban Suez - Egypt

**DOI:** 10.11604/pamj.2013.14.26.1609

**Published:** 2013-01-17

**Authors:** Mohammed Hany Kamel, Abdulmajeed Ahmed Abdulmajeed, Sally El-Sayed Ismail

**Affiliations:** 1 Faculty of Medicine- Suez Canal University, Ismailia, Egypt

**Keywords:** Falls, elderly, risk factors, primary care, assessment, causes

## Abstract

**Introduction:**

Falling is one of the most common geriatric syndromes threatening the independence of older persons. Falls result from a complex and interactive mix of biological or medical, behavioral and environmental factors, many of which are preventable. Studying these diverse risk factors would aid early detection and management of them at the primary care level.

**Methods:**

This is a cross sectional study about risk factors of falls was conducted to 340 elders in Urban Suez. Those are all patients over 60 who attended two family practice centers in Urban Suez.

**Results:**

When asked about falling during the past 12 months, 205 elders recalled at least one incident of falling. Of them, 36% had their falls outdoors and 24% mentioned that stairs was the most prevalent site for indoor falls. Falls were also reported more among dependant than independent elderly. Using univariate regression analysis, almost all tested risk factors were significantly associated with falls in the studied population. These risk factors include: living alone, having chronic diseases, using medications, having a physical deficit, being in active, and having a high nutritional risk. However, the multivariate regression analysis proved that the strongest risk factors are low level of physical activity with OR 0.6 and P value 0.03, using a cane or walker (OR 1.69 and P value 0.001) and Impairment of daily living activities (OR 1.7 and P value 0.001).

**Conclusion:**

Although falls is a serious problem among elderly with many consequences, it has many preventable risk factors. Health care providers should advice people to remain active and more research is needed in such an important area of Family Practice.

## Introduction

In Egypt, there is gradual increase in the absolute and relative numbers of older people over the last few decades. Older people defined as 60 years of age and more constituted 6.1% of the total population in 1996 and is expected to reach 8.9% in 2016 and 10.9% in 2026 [1]. Accordingly, the expected rate of average total population from 1996 to 2026 is about 57% while the expected rate of increase among older people during the same period is about 79% [2]. The last country profile of Egypt shows that the percentage of older people(more than 65 years) is 3.7% of the total population in 2009 [3].

The life expectancy for males at birth was 60.5 years in 1986 while for older people was 14.3 years. In 2026 the expected life expectancy for males at birth will be 74.7 years and for older people will be 19.3 years. So it is concluded that the percent of increase in life expectancy for males at birth from 1986 to 2026 = 23.5% and for older people = 35%. Similarly, for females at birth the percent increase = 25% and for older females = 44%. Again, this reflects the importance of providing health care for older people in Egypt [4].

The actual definition of a fall in older people has been open to some debate. A frequently used definition is "unintentionally coming to the ground or some lower level and other than as a consequence of sustaining violent blow, loss of consciousness, sudden onset of paralysis as in stroke or an epileptic seizure. This definition excludes overwhelming external disturbances that result in an older person being knocked over, and major internal disturbances that cause an older person to collapse instead of fall. Some researchers use a broader definition of falls to include those that occur because of dizziness and syncope [5].

A fall also can be defined as a sudden, unintentional change in position causing an individual to land at a lower level, on an object, the floor, or the ground, as a consequence of sudden onset of paralysis, epileptic seizure, or overwhelming external force [6].

Falling is a common serious medical condition that affects the health of elderly persons. Falling is a misdiagnosed problem in primary care with a substantial impact on healthcare costs. It is one of the most common geriatric syndromes threatening the independence of older persons [7]. Falls result from a complex and interactive mix of biological or medical, behavioral and environmental factors, many of which are amenable to intervention, most injuries are the result of preventable factors rather than random “accidents”. A meta-analysis of 74 studies about the risk factors of falls confirmed this multifactorial etiology with the strongest associations were found for history of falls (OR = 2.8 for all fallers; OR = 3.5 for recurrent fallers), gait problems (OR = 2.1; 2.2), walking aids use (OR = 2.2; 3.1), vertigo (OR = 1.8; 2.3), Parkinson disease (OR = 2.7; 2.8), and antiepileptic drug use (OR = 1.9; 2.7) [8].

Environmental hazards accounting for about 25 to 45% in most studies. One half to two thirds of falls occur in or around the patient's home [5].

As most of falls are associated with one or more identifiable risk factors (e.g. weakness, unsteady gait, confusion and certain medications), and research has shown that attention to these risk factors can significantly reduce rates of falling [9].

However, one study in Australia compared the effect of three different interventions on elderly over 70 and found that among individual interventions, exercise had the greatest impact on reducing falls. While home modifications or vision correction had a minimal reduction in the subjects annual fall rate of 3.1% and 4.4%, respectively, compared with the exercise group, which had a significant 6.9% reduction. The group who had all three interventions fared the best, with an estimated reduction in annual falls of 14% [10].

Despite the body of knowledge about the falls problem available in international literature, there are no available studies to describe the possible risk factors and consequences of such an important problem in the Eastern Mediterranean region.

## Methods

A descriptive cross-sectional study was held to assess the prevalence and risk factors of falls among elderly people. The study was conducted to persons above 60 who attended two urban primary health care centres in Suez City ? Egypt during the study period. All subjects have given a written consent to participate in this study. Those who did not agree to participate or who were severely ill were not included in this study.

**Sample size**: considering 30% prevalence of falls from other studies [4, 11], with 95% confidence level, our sample size was estimated to be 340 participants.

**Sampling method**: Continuous recruitment was used by including all elderly persons who fulfilled the inclusion criteria and attended for medical services in the two primary health care centers.

**Data Collection**: Every recruited participant was exposed to a questionnaire included:

1. History of any falling incident as memorized by the patient or his caregiver
2. Socio-demographic data
3. Risk factors of falls including use a cane or walker, falling during 12 months ago, acute or chronic illness, types and number of medications, physical deficits (balance and gait disorders, weakness, pain related to arthritis, visual and auditory impairment, epilepsy, parkinsonism, vertigo,, syncope, dizziness upon standing, foot problems, difficulty rising from a chair, fear of falling) [7].
4. Assessment of functional disability in activities of daily living using Katz instrument. The elderly takes 1 point for being independent in one activity and 0 points for being dependent. The scoring ranges from 6 that mean that the person has a full function to 2 or less which reflects severe functional impairment [12].
5. Home hazards [11]
6. Mini-mental state examination scale: this scale can be administered in 10 to 20 minutes. It consists of 11 items; the scoring falls in a range from 0- 30, a score of 23 or less correlates well with impaired cognitive function
7. Nutritional Health Checklist: It uses the mnemonic “determine” to assess a patient's risk of Scoring: 0-2 = good; 3-5 = moderate nutritional risk; 6 or more = high nutritional risk
8. Geriatric depression scale where normal individuals score from 0-5 while above 5 suggests depression
9. Clinical examination: BMI, Blood pressure, Gait and balance evaluation (Timed “Up & Go” test)

To ensure the validity of the used tools, 10 elderly participants were piloted by the data collectors and some modifications were done accordingly. The data collection process was conducted by the researcher and two trained community nurses through direct interview with elderly subjects.

## Results

The study included 340 participants of whom females represent (63.2%). More than half of the studied population were in the age group of 60-64; their percent were (53.5%), while elders more than 75 years were (6.8%). Most of the studied elders were married and more than half of them (54.1%) lived with their spouses and children. Almost half (45%) of the studied elders were illiterate (9.4% males and 35.6% females). As for their income source, they mostly depended on their pensions (91.3%).

The estimated prevalence of falls during the past 12 months among the study population is 60.3% (52.3% ? 69.1% at 95% confidence interval). One third of falls (36%) occurred outdoors, 24% occurred on stairs and (17.5%) occurred in bathrooms. As regards for the most common diseases among the elders who reported falls, about one third of them (30.0%) have diabetes mellitus, another third (29.7%) have hypertension and (18.5%) have osteoarthritis.

Table 1 summarizes univariate analysis between falls and several risk factors. All variables showed statistical significance with falls except for the gender despite the fact of Falls being more common among females than males. as Half of the elders with history of falls (45.9%) described themselves as inactive before the falling incident which was proved to be a statistically significant relationship and another significance was detected between falls and living alone as 12% of the fallers live alone compared to only 4.2% who live alone but did not report previous falling.

**Table 1:** Risk of falls among elderly according to different risk factors (Univariate analysis)

Variable		History of falls (n=205)	No history of falls (n=135)	Odds Ratio	95% C.I.	P-value
		No (%)	No (%)			
Gender	Male	74 (21.8%)	51 (15%)	1.07	0.69 – 1.69	0.7535
	Female	131 (38.5%)	84 (24.7%)			
Age	<70	153 (45%)	118 (34.8%)	2.36	1.3-4.29	0.005 *
	>70	51 (15%)	18 (5.2%)			
Physical Activity	Active	49 (14.4%)	74 (21.8%)	3.86	2.42 – 6.16	< 0.001 *
	Not active	156 (45.9%)	61 (17.9%)			
With whom does the elder live	with some one	164 (67.4%)	121 (35.8%)	2.16	1.13 – 4.14	0.02 *
	Alone	41 (12%)	14 (4.2%)			
Use a Cane or walker	Yes	59 (17.3%)	5 (1.5%)	10.51	4.09 – 26.98	< 0.001 *
	No	146(43%)	130 (38.2%)			
Medication use	Yes	174(51.2%)	93 (27.3%)	2.71	1.59 – 4.63	0.0003 *
	No	29(8.5%)	42(13%)			
Home hazards	Safe	95 (27.9%)	83 (24.5%)	1.83	1.18 – 2.85	0.007 *
	Unsafe	110 (32.3%)	52 (15.4%)			
Activities of daily living	Full function	129(37.9%)	127(37.3%)	9.35	4.34 – 20.17	< 0.001 *
	Impairment	76 (22.3%)	8 (2.4%)			
Mini mental state	Normal	95(27.9%)	85(25%)	1.97	1.26 – 3.07	0.003 *
	Cognitive impairment	110(32.3%)	50(14.7%)			
Geriatric depression scale	Normal	121(35.6%)	114(33.5%)	3.77	2.19 – 6.48	< 0.001 *
	Depressed	84(24.7%)	21(6.2%)			
Nutritional Risk	Good	16(4.7%)	34(10%)	3.98	2.09 – 7.55	< 0.001 *
	Risky	189 (55.6%)	111 (29.7%)			

(17.3%) of fallers who uses a cane or a walker reported falls incidents compared to only (1.5%) who uses them and did not report falls with a strong significance (Odds ratio (OR) 10.51 and P value <0.001). Using medications was significantly associated with falls. About one third of fallers had gait and balance disorders while (34.4%) of fallers had visual disorders and (11.8%) of them suffered from hearing problems. Almost all physical deficits were proved to be statistically significant with falls among studied elders. The percentage of those who reported falls is significantly higher among elders with some degree of impaired mobility in daily living activities (22.3%) compared to (22.4% in those who had some impairment in daily activities but reported no history of falls. (OR 9.35 and P value <0.001).

Having unsafe homes, being depressed, impaired cognitive function, and being at high nutritional risk are all significantly associated with falls. The relation between falls and mini mental state examination revealed that about (47.1%) of studied population had cognitive impairment and (32.3%) of them had a history of falls with another statistical significance. About 30.9% of studied population were depressed, and (24.7%) of them had a history of falls. Most of studied population (64.4%) are at high nutrition risk, and more of fallers (44.7%) had high nutrition risk, while only (4.7%) of fallers had good nutrition.

Table 2 is a multivariate logistic regression for predictors of falls proves that the strongest risk factors are low level of physical activity with OR 0.6 and P value 0.03, using a cane or walker (OR 1.69 and P value 0.001) and Impairment of daily living activities (OR 1.7 and P value 0.001)

**Table 2:** Multivariate logistic regression analysis for the predictors of falls among elderly

Risk Factors	Coefficient	P-value	Adjusted OR (95% CI)
Gender (Female / Male)	0.183	0.524	1.20 (0.68 – 2.11)
Age (≥70/ <70)	0.284	0.434	1.33 (0.65 – 2.71)
Physical Activity (Not active/ Active)	0.598	0.03*	1.82 (1.05 – 3.15)
With whom does the elder lives (Alone/ Not alone)	0.328	0.400	1.39 (0.65 – 2.98)
Use a Cane or walker (Yes/ No)	1.69	0.001*	5.42 (1.95 – 15.08)
Medication use (Yes/ No)	0.462	0.149	1.59 (0.85 – 2.97)
Home hazards (Unsafe/ Safe)	0.521	0.050	1.68 (1.0 – 2.83)
Activities of daily living (Impairment/ Full function)	1.736	< 0.001*	5.67 (2.44 – 13.21)
Mini mental state (Cognitive impairment/ Normal)	0.151	0.597	1.16 (0.67 – 2.03)
Geriatric depression scale(Depressed/ Normal)	0.475	0.147	1.61 (0.85 – 3.05)
Nutrition risk assessment (Risky/ Good)	0.692	0.064	2.0 (0.96 – 4.16)
*Constant*	- 2.07	< 0.001*	0.13

## Discussion

In the period from 8-2010 to 8-2011 340 perons above the age of 60 in Suez city - Egypt agreed to be enrolled in this study to determine the relationship between several risk factors and falls. 205 (60.3%) of the studied population reported at least one incident of falling. Other studies gave higher rates of falls [13, 14]. This might be explained by the presence of multiple risk factors among the studied population. Several studies have shown that the risk of falling increases dramatically as the number of risk factors increases.

In the current study, age was significantly related to falls as 15% of the persons above 70 reported at least one incident of falling during the last year compared to only 5.2% of those who are in the same age group and did not have falls. This finding is supported by other studies [11], while disagrees with others [15]. In addition, the present study indicated that falls were higher among females than males; this can be explained as most of studied elders were women as they are more frequent users of medical services. This result confirms with other studies [11, 16]. About one third of studied population who had falls was illiterate and there was no statistical significant difference between educational level and falls, this result agrees with by Reyes-Ortiz et al. [17]. However, it disagrees with a study conducted in USA [15].

More than half of the studied elders live with their families, and about 16.2% live alone with a significant difference regarding to falls. Some studies found no significant relation between living alone and falls [11, 18] while others proved that subjects living alone have an increased risk of falling of about 20-30% [17]. Another significance was proved between living in unsafe homes and falls among the study population. This was studied by Lord et al. who found that those who had one or more environmental hazards in their homes were more likely to have reported falling in the last 3 months [19]. Researchers at the Accident Research Center in Victoria, Australia, conducted that modification of home environment and correction of vision decreased the annual fall rate of study subjects [20].

More than one third of fallers in the current study experienced falls outside their houses (36%), followed by falling on stairs (24%) and in the bathroom (17.5%). These results are consistent with Li W et al [21]. When asked to describe the possible causes of their falls: most of fallers recalled dizziness (31%) as the cause of their falls followed by legs giving way (26%) and the least recognized cause was tripping (6%) while only 3.5% of fallers could not find a reason. In a study conducted in the UK, 53% reported tripping, 8% dizziness and 6% reported blackouts. 19% were unable to give a reason [22].

We found that there is a significant relation between falls and physical activity, as most of the studied elders are not used to exercise regularly and proved to be more prone to falls than others who were physically active and this is supported by some studies as well [11, 14]. A systematic review of risk factors for falls in older people found no clear association emerged with physical activity [17]. As shown in the present study, there is a strong relation between the use of walking aids and falls; this result is confirmed by Abdulmajeed et al. [11] as well as a systematic review conducted within the Apollo project [18].

There is a significant relation between falls among elderly and medications use as most (78.5%) of studied elders use medications; of them 51.2% had a history of falls. This may be explained by interactions resulting from polypharmacy as well as medications affecting alertness and coordination. About one third of the study population use NSAIDs and about half use hypoglycemic medications. Other studies disagree with our findings as antidepressants showed the strongest statistical association with falling followed by anti-psychotics/neuroleptics in a study examined the Impact of 9 Medication Classes on fall in Elderly Persons [23]. However, the lack of reliable recording system and the possible cognitive impairment may affect our study results which mainly depended on the elderly patients ability to recall and recognize the medications they are receiving.

The majority (75.3%) of elders in the current study have a highly functional status score However, there is a significant relation between falls and impairment in activities of daily living. This agrees with Abdulmajeed et al. who found that 87.6% were undependable for activities of daily living, but those who need help for activities of daily living were more exposed to falls [11]. The American Geriatrics Society, British Geriatrics Society, and American Academy of Orthopaedic Surgeons confirmed that impaired activities of daily living is one of the high-risk situations that can cause or contribute to falls [24].

Depression and falls were significantly associated in this study as 24.7% of depressed patients suffered from falls (OR. 1.97; 95% CI: 2.19-6.48) which is consistent to Cavanillas et al. who proved that depression (OR. 3.3; 95% CI: 1.8±6.1) represented a significant risk only for falls with intrinsic precipitating causes [25].

In the present study (64.4%) most of studied elders have some degree of nutritional risk; of them 66.6% had history of falls and this relation is significant and goes with the systematic review conducted within the Apollo project which concluded that malnutrition is a potentially relevant factor, which has been neglected by researchers so far [19].

In the present study, it is shown that most of studied elders had variable mobility, and the risk of falling increases with impaired mobility(OR. 3.86; 95% CI: 2.42-6.16). This is consistent with Fletcher and Hirdes who determined that seniors with impaired mobility were 1.65 times more likely to experience a fall, as compared to those with no impairments in mobility [26].

In a meta analysis that included 74 studies that examined 31 risk factors [27], the strongest associations were found for history of falls, gait problems, using walking aids (OR = 2.2; 3.1) this last risk factor is also found as one of the strongest in our study according to the regression analysis with OR = 1.69. Other factors reported by this meta analysis either were not examined in the current study or showed no significance. This can be to the different tools used to assess those risk factors.

## Conclusion

This study provides evidence that falls is a common problem among elderly people with numerous preventable risk factors. Routine comprehensive geriatric assessment whenever possible is important to detect possible factors that might be associated with falls such as: impaired daily living activities and general inactivity as health care providers should encourage old people to remain active. Thus, there may be good reasons to expect benefits from preventive measures addressing individual fall risk factors but more research is required to understand under which conditions these expectations are realistic. The magnitude of the problem in Eastern Mediterranean countries also needs to be addressed.

### Limitations

In availability of a reliable record system in the study setting prevented the researches from having more accurate data about history of falls and other risk factors that the patient might not recall. Thus, it cannot be ruled out that some conditions, e.g. loss of independency in ADL or in physical activity, were rather a consequence of falling than a predictor. Overall, the results give some hints which factors may be strongly associated with fall accidents in the Egypt elderly population but they provide no robust prove of etiology of these incidents. A prospective observational study would better suit this purpose.
